# The Effects of N-acetylcysteine on Transient Receptor Potential Melastatin 2 Channels Activation and Expression in Testicular Tissue of Diabetic Rats

**DOI:** 10.7759/cureus.38661

**Published:** 2023-05-07

**Authors:** Ahmet Turk, Mustafa Ulas, Abdullah Karadag, Nevin Kocaman, Ebru Onalan, Tuncay Kuloglu

**Affiliations:** 1 Department of Histology and Embryology, Faculty of Medicine, Adiyaman University, Adiyaman, TUR; 2 Department of Physiology, Faculty of Medicine, Firat University, Elazig, TUR; 3 Department of Physiology, Faculty of Medicine, Adiyaman University, Adiyaman, TUR; 4 Department of Histology and Embryology, Faculty of Medicine, Firat University, Elazig, TUR; 5 Department of Medical Biology and Genetics, Faculty of Medicine, Firat University, Elazig, TUR

**Keywords:** diabetes mellitus, n-acetyl cysteine, oxidative stress, stz, trpm2

## Abstract

Introduction: Diabetes mellitus (DM) is a common, chronic metabolic disease that has harmful effects on many diverse tissues, including the testis. One of the ways of tissue damage is the modification of transient receptor potential melastatin 2 (TRPM2) channels by increased reactive oxygen species (ROS). In our study for the first time, it was aimed to investigate TRPM2 channel activation in testicular tissues of diabetic rats induced by streptozotosin (STZ) and to examine the efficacy of N-acetylcysteine (NAC) treatment, which is an antioxidant.

Methods: In our study, 28 Wistar albino male rats aged 8-10 weeks were used, and animals were divided into four groups: control group, NAC group, DM group, and DM + NAC group. The experimental phase was designed as eight weeks. The malondialdehyde (MDA) level, which is an indicator for lipid peroxidation due to oxidative stress, was measured by the spectrophotometric method. The Tunel assay was used to determine apoptosis on testicular tissue. TRPM2 immunoreactivity was determined by the avidin-biotin-peroxidase complex method, and quantitative polymerase chain reaction (QPCR) was used to determine TRPM2 expression levels.

Results: It was seen that MDA levels were significantly increased in the DM group and decreased after NAC treatment. Similarly, it was observed that apoptosis levels, which increased significantly in diabetic rats, decreased to the levels of the control group after treatment. It was seen that TRPM2 activation and expression levels were significantly decreased in the DM group.

Conclusion: The results of this study show that NAC regulates TRPM2 activation in the testicular tissue of patients with diabetes and has tissue-protective properties.

## Introduction

It has been reported that infertility rates increase in men because of increasing type I and type II diabetes in developed countries [1]. Studies conducted on diabetic patients have shown that penile erection, ejaculation, and spermatogenesis are negatively affected. Besides, it has been shown that increased free radicals play an important role in the pathogenesis of diabetes by causing cell death and tissue damage [2]. Therefore, increased apoptosis causes dysfunction in the testicles of diabetic patients [3]. In studies on rats with diabetes mellitus (DM) induced by streptozotosin (STZ), it has been reported that follicle-stimulating hormone (FSH) synthesis, testosterone levels, and luteinizing hormone (LH) levels decreased owing to the negative effects of diabetes on the receptors (FSH, insulin, and insulin-like growth factor) of the seminiferous tubule [4].

Reducing oxidative stress-related tissue damage in DM is one of the therapeutic strategies, and there are a limited number of papers on the male reproductive system [5]. N-acetylcysteine (NAC), a glutathione (GSH) precursor, replenishes glutathione (GSH) stocks, increases superoxide dismutase (SOD) activity, reduces hydroxyl radicals, and inhibits autocatalytic lipid peroxidation [6]. It provides antioxidant activity by regulating GSH levels, directly scavenging free radicals and suppressing neutrophil activity and tumor necrosis factor (TNF) production [7]. Therefore, it stands out as an ideal antioxidant agent in experimental studies.

Transient receptor potential melastatin 2 (TRPM2), which is a multifunctional, Ca^+2 ^permeable, non-selective cation channel, is detected in many tissues, including pancreatic β-cells, but is highest expressed in the brain [8]. It is shown that TRPM2 plays a key role in the entry of Ca^+2^ ions in cells that depolarize due to insulin release in β-pancreatic cells [9]. Furthermore, since TRPM2 can be activated by oxidative stress, it has recently been seen as a potential therapeutic target against oxidative stress-related diseases, including diabetes, inflammation, myocardial infarction, and neurodegenerative diseases [10].

In our study, we aimed to investigate the effects of NAC treatment on apoptotic changes and TRPM2 channel expression in experimentally induced rat testicular tissues.

This article was previously posted to the Research Square preprint server on April 27, 2022.

## Materials and methods

Animals

In our study, 28 Wistar albino male rats, 8-10 weeks old and weighing 250-270 g, were used. Animals were divided into four groups (seven in each group) and had ad libitum access to food and water. They were kept under a photoperiod of 12 hours of light and 12 hours of darkness in controlled temperature conditions of (20-240°C). The experimental phase was designed as eight weeks.

*Control Group* 

No action was taken during the experiment phase.

NAC Group

About 100 mg/kg of NAC was administered intraperitoneally (i.p.) every day during the experimental period.

DM Group

A single dose of 50 mg/kg STZ was dissolved in 0.1 M sodium citrate buffer (pH: 4.5) and administered i.p. After 72 hours, blood samples were taken from the tail veins of rats that had fasted for 12 hours. Blood glucose was measured in a glucometer device, and rats whose fasting blood glucose level exceeded 250 mg/dl were considered diabetic.

DM + NAC Group

In addition to the DM group, 100 mg/kg NAC was administered i.p. every day during the experimental period.

At the end of the experiment, rats in all groups were anesthetized by i.p. administration of ketamine (75 mg/kg) + xylazine (10 mg/kg), and testicular tissues were rapidly removed. Tissue samples were stored under suitable conditions for histological and biochemical studies.

Malondialdehyde (MDA) levels

Firstly, the tissues were homogenized. Then it was centrifuged, and the supernatant was taken into another 1 ml tube. One milliliter of 10% trichloroacetic acid (TCA), 1 ml of 0.6% 2-thiobarbituric acid (TBA), 1 ml of distilled water, and 0.5 ml of 4% HCl were added to the tube and mixed. The prepared mixture was incubated at 90-95°C for 120 minutes. After incubation, 3 ml of butanol was added and vortexed. After that, the tubes were centrifuged at 5000 rpm for 5 min, and the butanol phase was read at 532 nm against butanol in the spectrophotometer. The absorbance value (X) was calculated with the formula ((X + 0.0344)/0.0492). The value found was multiplied by 5 (because tissue homogenate was prepared in 5 ml buffer).

Determination of apoptosis

The Tunel method was used to determine apoptosis in testicular tissue. Sections of 5-6 μm thickness from paraffin blocks were taken on poly-l-lysine slides. Cells undergoing apoptosis were determined using the ApopTag Plus Peroxidase In Situ Apoptosis Detection Kit (Chemicon, Cat No. S7101, USA) in accordance with the manufacturer's instructions. Preparations were examined and photographed under the Novel N-800M microscope (Novel, China). In staining with Harris hematoxylin, blue-stained nuclei were considered normal, and brown-stained nuclei were considered apoptotic. At least 500 normal and apoptotic cells were counted under 10× magnification in randomly selected areas. The apoptotic index (AI) was calculated by dividing the apoptotic cells by the total number of cells. Scale bar: 50 µm.

Determination of TRPM2 immunoreactivity

The avidin-biotin-peroxidase complex method was applied to determine TRPM2 immunoreactivity in testis tissue. Deparaffinized tissues were incubated with a primary antibody (Rabbit Anti-TRPM2 antibody, ab101738, Abcam, Cambridge, UK) for 60 minutes. Then the tissues were incubated with a secondary antibody (biotinylated goat anti-polyvalent (anti-mouse/rabbit IgG), TP-125-BN, Lab Vision Corporation, USA) for 30 minutes in a humid environment at room temperature. The Fast Red Substrate System (TA-125-AF, Lab Vision Corporation, USA) solution was dripped onto the tissues. Then staining was done with Mayer's hematoxylin and observed under a light microscope. Positive and negative controls were made according to the manufacturer's instructions. The preparations were examined and photographed under the Novel N-800M microscope. The evaluation of immunohistochemical staining was based on the intensity of staining. The intensity of immunostaining was scored semi-quantitatively with numbers from 0 to +3.

RNA isolation

PureLink™ RNA Mini Kit (Thermo Fisher Scientific Inc., Waltham, MA, USA) was used for RNA isolation from testicular tissue. A lysis buffer solution was obtained by mixing 1 ml of lysis buffer and 10 μl of 2-mercaptoethanol in the falcon tube. Testicular tissue, homogenizer beads, and 600 µl of lysis buffer solution were placed in Eppendorf tubes. The tissues were lysed in a homogenizer for six minutes and then centrifuged at 12,000×g for two minutes at room temperature. All of the RNA-containing liquid phases were taken to a new Eppendorf tube, and isolations were performed according to the recommended protocols of the manufacturer. Total RNA was stored at −80 °C until use.

cDNA synthesis

In our study, the Qubit® RNA Assay Kit (Thermo Fisher Scientific Inc., Waltham, MA, USA) was used for RNA measurement. RNA amounts were measured spectrophotometrically and equalized for cDNA synthesis. cDNA synthesis was carried out using 10 µl of RNA sample, 2 µl of 10XRT random primer, 2 µl of 10XRT buffer, 0.8 µl of 25XdNTP mix, 4.2 µl of nuclease-free water, and 1 µl of MultiScribe™ Reverse Transcriptase Enzyme in thermal cycler device. cDNA samples were stored at −20°C until use.

Real-time-polymerase chain reaction (PCR)

For the determination of TRPM2 expression levels, TRPM2 primer (Catalog No. Rn-01429417-m1) and TaqMan Master Mix were used, and reactions were implemented in the Applied Biosystems 7500 Real-Time PCR system. For the normalization of mRNA expression levels, glyceraldehyde-3-phosphate dehydrogenase (GAPDH) (Catalog No. Rn-01775763-g1) was used as a housekeeping gene.

Statistical analysis

Obtained data were determined as mean ± standard deviation. GraphPad software (GraphPad Prism version 8.0.0 for Windows, GraphPad Software, San Diego, CA, USA) was used for statistical analysis. Groups were analyzed using one-way analysis of one variance (ANOVA) and the post-hoc Tukey test. p<0.05 values were considered statistically significant.

## Results

MDA levels

**Figure 1:**
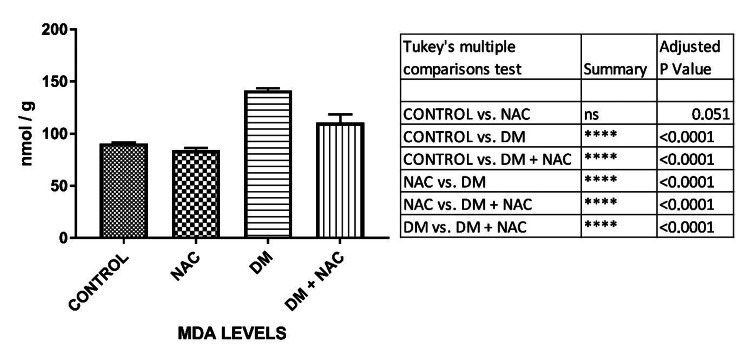
Analysis of malondialdehyde levels measured by the spectrophotometric method in testicular tissues. The differences between the groups are given in the table. MDA: malondialdehyde, NAC: N-acetylcysteine, DM: diabetes mellitus.

MDA levels measured in the testicular tissues of the control and NAC groups were found to be similar. MDA levels were significantly increased in the DM group compared to the control and NAC groups (p<0.0001). It was seen a significant decrease in the DM + NAC group according to the DM group (p<0.0001) (Figure 1).

Tunel assay

**Figure 2:**
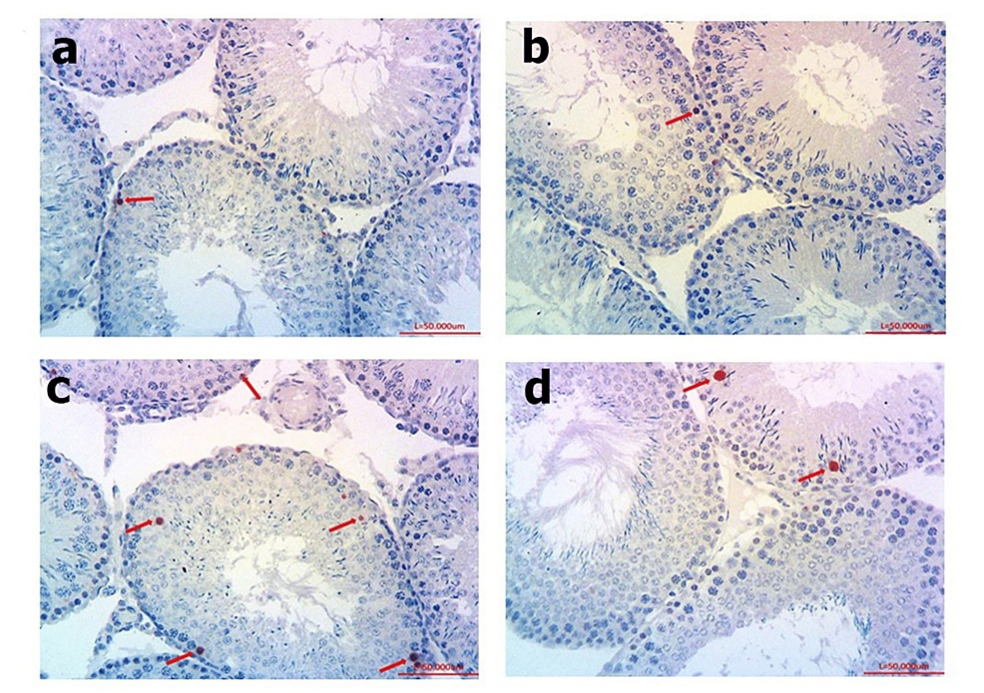
Representative photomicrographs of Tunel staining in the testes. (a) Control group, (b) NAC group, (c) increased apoptotic cells of DM group, and (d) decreased apoptotic cells of DM + NAC group. Red arrows indicate candidate apoptotic cells. Scale bar: 50 µm. NAC: N-acetylcysteine, DM: diabetes mellitus.

**Figure 3:**
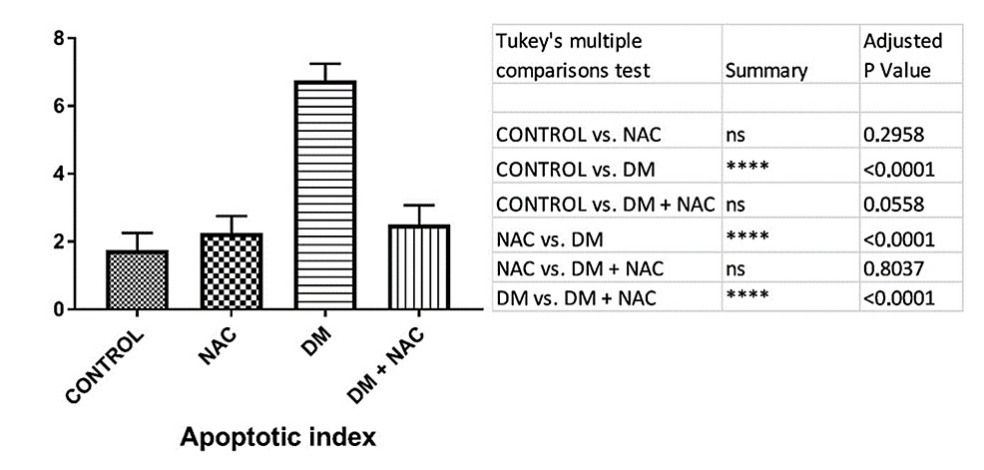
The results obtained by analyzing the apoptotic index on the testicular tissue. NAC: N-acetylcysteine, DM: diabetes mellitus.

Images that were obtained under the light microscope by staining the testicular tissue with the Tunel method are shown in Figure 2. It was shown that the apoptotic indexing of Tunel-positive cells of testes tissues between groups in Figure 3.

TRPM2 immunoreactivity

**Figure 4:**
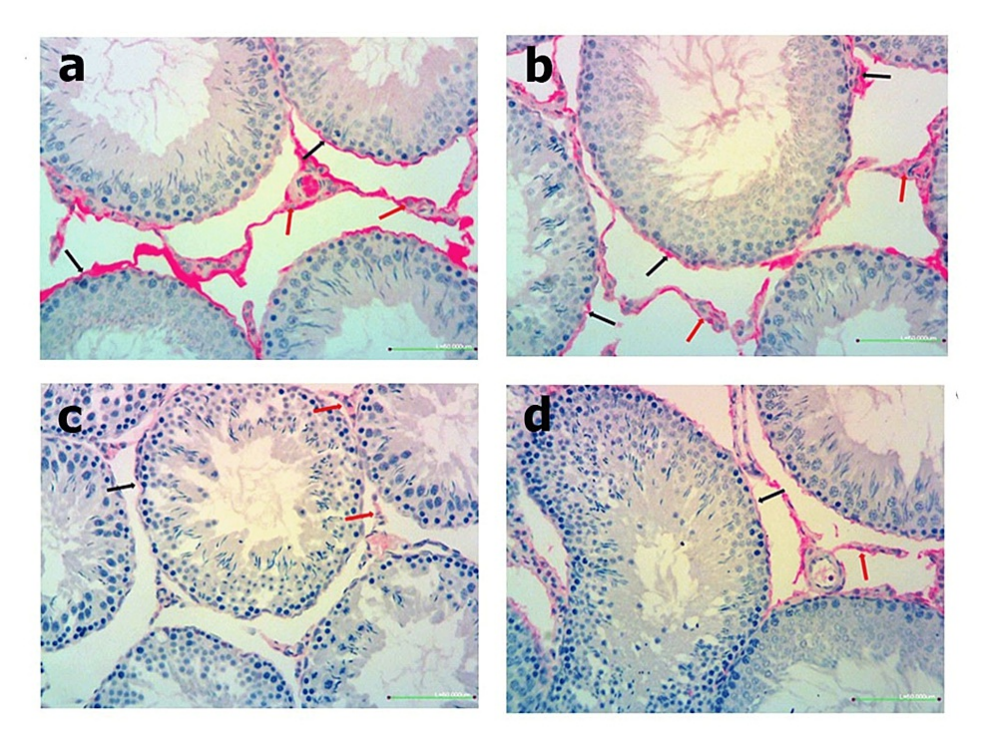
Immunohistochemical staining for TRPM2 (red arrow) in testis tissue. (a) Control group. (b) NAC group. (c) Decreased TRPM2 immunoreactivity of DM group. (d) Increased TRPM2 immunoreactivity of DM + NAC group. Scale bar: 50 µm. NAC: N-acetylcysteine, DM: diabetes mellitus, TRPM2: transient receptor potential melastatin 2.

**Figure 5:**
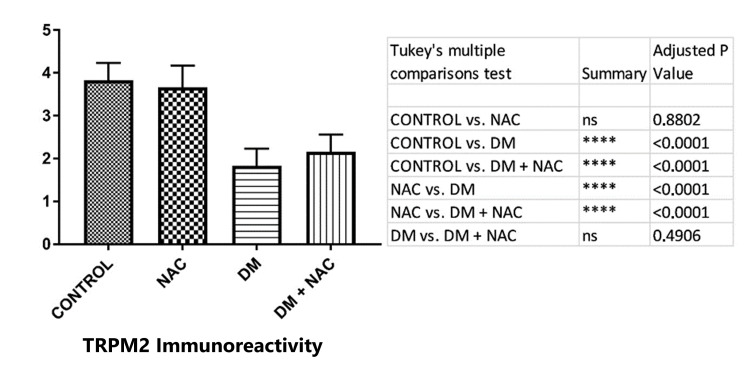
The results obtained by analyzing TRPM2 immunoreactivity on the testicular tissue. NAC: N-acetylcysteine, DM: diabetes mellitus, TRPM2: transient receptor potential melastatin 2.

Images that were obtained under the light microscope by immunohistochemical staining of testicular tissue in order to determine the immunoreactivity of TRPM2 (Figure 4). Figure 5 shows the histoscoring of the TRPM2 immunoreactivity of testes cells.

TRPM2 expression

**Figure 6:**
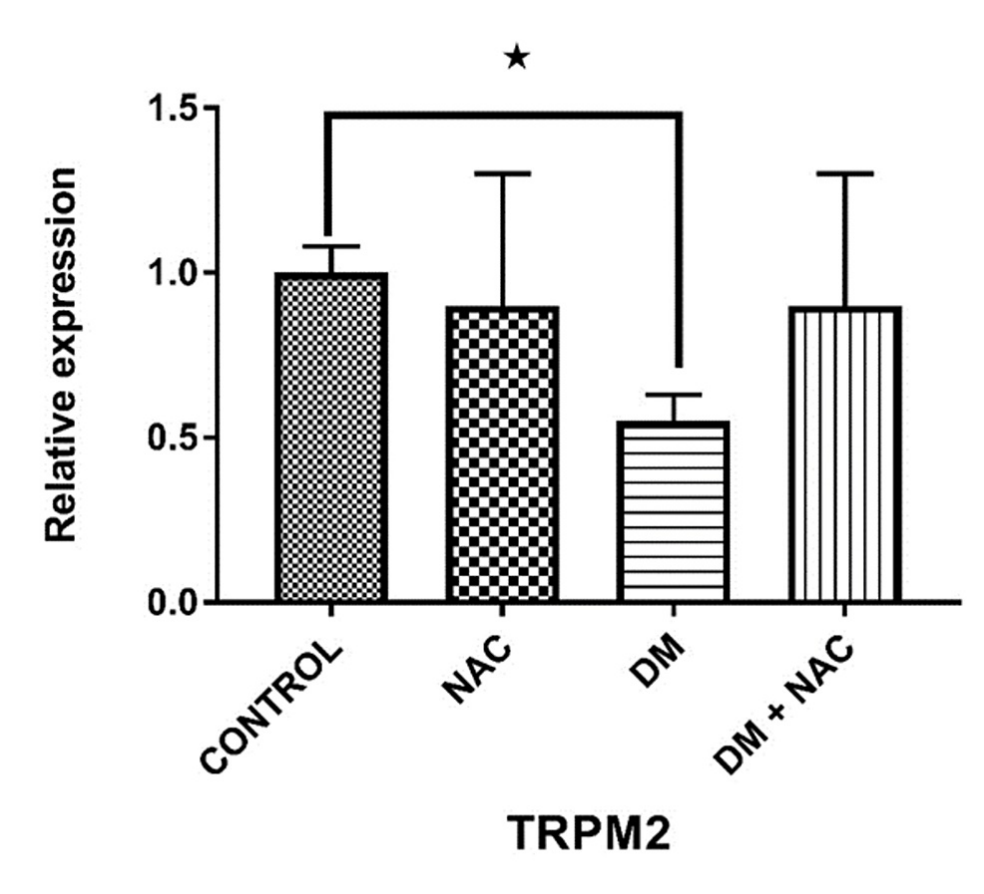
TRPM2 expression levels in testicular tissues. NAC: N-acetylcysteine, DM: diabetes mellitus, TRPM2: transient receptor potential melastatin 2.

Analysis of TRPM2 expression levels in testicular tissues showed a significant decrease in the DM group compared to the control group (p=0.0355). Although a prominent increase was observed in the DM + NAC group, there was no significant difference according to the DM group (p=0.1337) (Figure 6).

## Discussion

DM is a multifactorial, chronic metabolic disease. It has effects on the male reproductive system, such as reduction of hormones (FSH, LH, and testosterone), harm to testicular tissue and seminiferous tubules, and deterioration of sperm count and morphology [11]. In our study, we aimed to examine both oxidative stress-induced tissue damage and the levels of TRPM2 channels, after NAC treatment in testicular tissue after the eight-week diabetic period. To our knowledge, this is the first study to demonstrate TRPM2 channel activity and level on diabetic testicular tissue.

It was observed that free oxygen radicals and lipid peroxidation are significantly increased in experimentally diabetic rats and diabetic patients. Therefore, oxidative stress is considered an important factor in the etiology and progression of DM. NAC, an important glutathione precursor, was used in our study because it is a therapeutic strategy used to reduce oxidative stress [12]. MDA is an indicator of lipid peroxidation due to oxidative stress. Similar to other studies, we found a highly significant increase in the DM group [5,13]. It was still found to be significantly higher than the control group, although there was seen a decrease in the MDA levels after the treatment.

An increased number of apoptotic cells is an indicator of tissue damage. In our study, it was found that the number of apoptotic cells increased significantly in the DM group, which was similar to other papers on the liver [14], pancreatic β cells [15], and seminiferous tubules [16]. After the treatment, cell death was decreased in the DM group, and the significant difference with the control group disappeared. This data reveals the long-term tissue-protective effect of NAC.

TRPM2 activation has been associated with insulin secretion and cell death in pancreatic β-cells [17]. In our study, TRPM2 activation and expression were found to be significantly lower in the DM group. Although TRPM2 expression levels approached normal after NAC treatment, it was observed a little increase in TRPM2 activation and no significant difference according to the control group. In previous studies conducted in the hippocampus [18], dorsal root ganglia and brain [19], and lungs [20] of diabetic rats, contrary to the findings of our study; TRPM2 levels were increased. The main difference between our study from the others is that the experimental period was eight weeks. In other studies, this period was a maximum of four weeks. Since more tissue damage has occurred in our samples over time, it is possible that TRPM2 levels have decreased. Another short-term study is needed to confirm that.

However, there are some limitations in this study. In order to elucidate the effects of TRPM2 on apoptotic pathways, its full activation should be monitored with a longer experimental process. In addition, it is necessary to evaluate the effects of NAC on this cation channel by applying antagonist treatment of TRPM2. We believe that the data to be obtained from studies to be conducted considering these limitations can offer clinicians and researchers a new and effective perspective for the use of NAC as a supplemental agent in the treatment of DM, one of the leading metabolic diseases of our age.

## Conclusions

In this study, it was observed that apoptotic cells developing due to increased oxidative stress in diabetic testicular tissue increased, whereas NAC, which is a glutathione precursor given as a treatment, suppressed apoptotic pathways and decreased the number of Tunel-positive cells. It has also been observed that NAC regulates TRPM2 activation in the early stages of diabetes. Therefore, our study shows that NAC regulates TRPM2 activation in the testicular tissue of patients with diabetes and has tissue-protective properties.
